# Antioxidant effect of gallic acid on retinal ganglion cells in glaucoma model

**DOI:** 10.1038/s41598-024-65965-7

**Published:** 2024-06-28

**Authors:** Ruping Jiang, Yao Lv, Binlin Chen, Xia Wu, Yuan Zou, Liang Liang

**Affiliations:** Department of Ophthalmology, Yichang Central People’s Hospital, The First College of Clinical Medical Science, China Three Gorges University, Yichang, 443003 China

**Keywords:** Gallic acid, Oxidative damage, Retinal ganglion cells, Acute ocular, Hypertension model, Diseases, Health care, Medical research

## Abstract

To evaluate the protective effect of gallic acid on the optic nerve by studying the inhibitory effect of gallic acid on oxidative stress in retinal ganglion cells. 100 male SD rats were randomly divided into four groups: normal control group, simple high IOP group, 0.5% gallic acid experimental group, and 1% gallic acid experimental group. HE staining, immunofluorescence, DHE staining, Western blot, and q-PCR were used to observe the antioxidant effect of gallic acid on the retina of acute ocular hypertension rats. HE staining of the retina of SD rats confirmed that the nucleus of RGCs was clear, the thickness of the RNFL was regular in the normal control group, and the nucleus of RGCs was ruptured and lysed in the simple high intraocular pressure (IOP) group and the gallic acid group, and the thickness of the RNFL was significantly thickened, but the thickness of the RNFL in the gallic acid group was significantly reduced compared with that in the simple high IOP group (p < 0.05). DHE staining showed that ROS content in the simple high IOP group was significantly increased compared with the normal control group, and ROS content was significantly decreased after the application of gallic acid (p < 0.05). Immunofluorescence staining with Brn-3a antibody confirmed that the number of RGCs was significantly reduced in the simple high IOP group compared with the normal control group, whereas after application of gallic acid, the number of RGCs was significantly more in the gallic acid group than in the simple high IOP group (p < 0.05). Western Blot and q-PCR confirmed that hypoxia-inducing factor 1α (HIF-1α) protein content and transcription level were significantly increased in the retinal tissue of the simple high IOP group, and gallic acid could inhibit HIF-1α protein content (p < 0.05) and reduce transcription factor level (p < 0.05). Gallic acid exerts a protective effect on RGC by inhibiting oxidative stress in rats with acute IOP elevation.

## Introduction

Glaucoma is a group of clinical syndromes characterized by optic nerve atrophy and visual field damage^1^. Its pathological feature is the apoptosis of Retinal Ganglion Cells (RGCs) caused by increased intraocular pressure. Pathological increase in intraocular pressure(IOP) is the main risk factor for glaucoma^2^. So far, the clinical intervention has mainly been limited to reducing IOP, but lowering IOP is not enough to prevent or reverse the progression of vision loss, and some patients still have further optic nerve atrophy and eventually blindness after controlling IOP to an ideal state^3^. Glaucoma is a disease involving many pathologic mechanisms, among which oxidative stress is one of the most important pathologic mechanisms^4^. Therefore, finding a drug with an antioxidant effect and protection of optic ganglion cells is the key to the treatment of optic nerve damage caused by glaucoma.

Gallic acid (GA), also known as 3,4,5-tri hydroxybenzoic acid, is a natural secondary metabolic product that can be isolated from wild Pueraria, Chinese medicine gallnut, and Malacca Puja. GA has one benzene ring, one carboxyl group, and three hydroxyl groups, and its unique chemical structure determines that it has a strong antioxidant function^5^. In vitro, antioxidant experiments of GA showed that gallic acid could effectively scavenge free radicals, hydroxyl free radicals, and superoxide ion free radicals, had obvious inhibitory effects on lipid peroxidation, strong reducing power, and total antioxidant power, and showed a good dose-dependent effect. It has been reported that the anti-oxidative stress function of gallic acid is inseparable from its unique chemical structure, and the phenolic hydroxyl group of gallic acid can provide hydrogen ions and ROS reactions generated during oxidative stress, remove ROS, and prevent the excessive formation of oxygen free radicals^6^. Gallic acid showed antioxidant effects by increasing the activity of cellular antioxidant enzymes such as hydrogen peroxide (CAT), glutathione peroxidase (GPx), and superoxide dismutase (SOD)^7^.

Hypoxia-inducible factor-1α (HIF-1α) is a major regulator of oxygen ion homeostasis. It is an oxygen-dependent transcriptional activator that increases its expression under hypoxia conditions and activates a series of target genes, which play an important role in many aspects, including neovascularization, apoptosis, and erythropoiesis^8^. The increase in HIF-1α is due to an increase in reactive oxygen species (ROS) produced by xanthine oxidase, leading to subsequent activation of HIF-1α protein synthesis by mammalian target rapamycin (mTOR) and decreased proline hydroxylation^9^. HIF-1α has been shown to increase immune markers in the optic nerve and retina in glaucoma compared to normal eyes. It has also been found that ROS is involved in the regulation of HIF-1α by intracellular silenced information regulatory factors^10^. Mice with silenced information regulatory factor gene knockout showed increased ROS levels and HIF-1α expression, indicating that ROS is closely related to HIF-1α. Under hypoxia conditions, ROS production in cell mitochondria is induced and HIF-1α is activated^9^. A large number of studies have proved that ischemia and hypoxia are the pathophysiological bases of optic nerve damage in glaucoma^11^.

No experiment has yet confirmed whether gallic acid can protect the optic nerve through antioxidant protection. Therefore, the aim of this experiment was to verify whether gallic acid can act as an optic nerve protector through the mechanism of antioxidant damage, and to provide an experimental basis for the clinical use of gallic acid to resist optic nerve damage in glaucoma.

## Materials and methods

### Main reagents

Rabbit poly anti-BRN-3A antibody (Abcam); HRP labeling of sheep and rabbit secondary antibodies (Wuhan Bude Company); ROS Dye Solution (Shanghai sigma-Aldrich); Rabbit monoclonal antibody HIF-1α (Chengdu Zheng’neng Company); DAPI dyeing reagent (Wuhan servicebio Company); RNA extract (Wuhan servicebio Company); Primer (Wuhan servicebio Company);Gallic acid (Shanghai selleck Company HPLC > 98%).

### Experimental animals

Specific Animal SPF(pathogen-free) 100 SD male mice purchased and fed from Laboratory Animal Center of Three Gorges University, Laboratory Animal production License No. SCXK(E)2022-0061. All experimental mice weighed 220-250g. The eyes of all experimental animals showed a normal red appearance, corneal transparency, normal anterior chamber depth, normal optometry, and normal fundus results. All animal procedures were carried out in line with the statement on the use of animals in ophthalmology and vision research issued by the Association for Research in Vision and Ophthalmology(ARVO). The animals were raised in the Barrier Laboratory of Animal Laboratory Center, China Three Gorges University, and approved by the Ethics Committee for Animal Experiments (No. 202205010I).

### Modeling and grouping

100 SPF-grade SD male rats were randomly divided into a normal control group,the simple high IOP group, and 0.5% gallic acid experimental group and 1% gallic acid experimental group, each group of 25 mice, each mouse weight 200–250 g. The 0.5% gallic acid group and the 1% gallic acid group were respectively given 30 mg/kg and 60 mg/kg gallic acid solution daily, for 7 consecutive days. During the period, the weight changes and eye changes of mice were recorded every day, and each group of mice ate freely. The rats were weighed and anesthetized by intraperitoneal injection of 2% pentobarbital sodium at 50 mg/kg. The surface of the eyeball was injected with proparacaine hydrochloride eye drops to perform the surface anesthesia of the conjunctival cornea, and then the compound tropicamide eye drop was applied to dilate the pupil of the rat under the operating microscope. Hang the infusion bag of 500 ml 0.9% sodium chloride injection on the infusion rack, and keep the difference between the liquid level of the infusion bag and the eye height of the rat about 150 cm (this height can form 110 mmHg intraocular pressure in the eye). The infusion tube is connected to a 4.5 gauge needle. After the dilation of the eyes of the rat was completed, the rat was lying on its side and the neck was covered with two folded gauze pieces, so that the head was fully exposed and the needle was easily fixed. The left hand opened the eyelid to make the eyeball slightly convex and fully exposed the eyeball and the limbus; the right hand placed needle No.4.5 with the infusion device at the temporal limbus of the eyeball of the rat horizontally into the needle, and the needle beveled upward. After penetrating the cornea, aqueous humor could be observed to flow out and the anterior chamber disappeared. Secure the needle with tape. During the perfusion process, it was observed that the cornea of the rat was dry and normal saline could be dripped. At this time, it could be observed under the operating microscope and direct ophthalmoscope. The conjunctival color of the eye of the rat was pale, corneal edema, fundus retina color was pale, and blood vessels were severed, indicating the formation of acute intraocular hypertension. After 1 h of maintenance, slowly reduce the height of the infusion bag to avoid bleeding and other reactions caused by rapid changes in intraocular pressure. Finally, when the fluid level in the infusion bag is reduced to the same level as the eyeball, pull out the infusion needle.

Follow up with levofloxacin eye drops to prevent infection. After the operation, the rats returned to the animal house when they were awake.

### Hematoxylin–eosin staining, retinal morphological observation and RNFL thickness measurement

Ten eyes were taken from each group and placed in eyeball fixation solution for 24 h for tissue fixation and were placed in a paraffin embedding machine, and an automatic embedding machine program was set. Remove the paraffin blocks after they have completely solidified. Dry overnight in an oven at 60 ℃, then dewaxing and hydration procedures were carried out, and continuous slices of about 5 μm in thickness were made within 2 mm of the front and back of the optic nerve as a sign. After dewaxing and dehydration, hematoxylin was stained for 10 min, 0.5% eosin solution for 2 min, 85%, 90%, 95%, and anhydrous ethanol (5 min each) were dehydrated, xylene was transparent and sealed with neutral glue, and photographs were taken under the microscope. ImageJ-i53 analysis software was used to measure the thickness of the inner limiting membrane from the nerve fiber layer to the junction of retinal ganglion cells.

### The number of surviving RGCs was detected by the immunofluorescence staining

Paraffin sections were prepared for ten eyeballs in each group, and dewaxing, dehydration, and antigen repair were performed in sequence according to the steps of the immunofluorescence experiment. The serum was sealed for 30 min, rabbit monoclonal Brn-3a antibody was incubated at 4 ℃ overnight, and then 1:500 diluted goat anti-rabbit secondary antibody covering tissue was added later, and incubated at room temperature for 50 min away from light. After DAPI restaining the nucleus, a self-fluorescence quenching agent was added for 5 min, and the slices were slightly dried and sealed with anti-fluorescence quenching sealing tablets. The sections were observed under a fluorescence microscope and the images were collected. The sections were observed under a fluorescence microscope and the images were collected. After the imaging was completed, the number of GCL layer positive cells in three fields of each section was measured using ImageJ-i53 analysis software.

### The ROS levels were detected by dihydroethidium staining

After removing the eyeballs of ten eyes in each group, the eyeballs were put into the frozen storage tube without any liquid and stored in the refrigerator at −80 ℃. After the eyeballs were removed, the tissues were immersed in an OCT embedding agent and frozen in liquid nitrogen. The frozen tissue was put into the frozen microtome and cut into slices with a thickness of 5–10 μm. The frozen slices were rewarmed at room temperature and the moisture was controlled. Use a tissue pen to draw circles around the tissue and add a self-fluorescence quencher for 5 min. Add ROS dye solution to the ring and incubate at 37 ℃ in a dark incubator for 30 min. Add DAPI dye solution and incubate at room temperature for 10 min away from light. Anti-fluorescence quenching sealing tablets. Image acquisition under fluorescence microscope: DAPI excitation wavelength is 330–380 nm, and the emission wavelength is 420 nm. After the imaging was completed, the number of GCL layer positive cells in three visual fields of each section was measured using ImageJ-i53 analysis software.

### HIF-1α protein level was detected by western blot

After sampling, retinal histamine was extracted, and equal amounts of protein samples(40 μL) were loaded onto 10% SDS-PAGE gel successively. Then, electrophoresed and transferred to nitrocellulose membranes, and which were blocked with 5% non-fat blocking grade milk were performed successively according to the steps of the Western-Blot experiment. Rabbit monoclonal HIF-1α antibody, rabbit monoclonal GAPDH antibody, and diluent were diluted at the ratio of 1:1000 and flooded on the closed PVDF membrane. Incubate at 4 ℃ overnight. Then the HRP-labeled goat anti-rabbit antibodies were diluted with a sealing solution at the 1:500 ratio, and incubated at room temperature for 1 h. Immunoblots were then visualized with an ECL Plus chemiluminescence reagent kit and quantified with optical methods using the ImageJ-i53 software. The results were normalized using GAPDH as an internal control.In order to save cost, when transferring the membrane at the end of the gel run, we cut the gel of non-destination proteins, leaving only the gel of the destination protein interval for transferring the membrane.

### HIF-1α relative transcriptional levels were detected by quantitative real-time polymerase chain reaction

RT-qPCR was used to detect the mRNA expression levels of HIF-1α in retina tissues. Reactions were run on a real-time PCR system according to the following protocol: 10 min at 55 °C, 1 min at 95 °C, followed by 40 cycles of 10 s at 95 °C and 35 s at 60 °C. Finally, the results were processed by ΔΔCT method, A = CT(target gene of experimental group) − CT(internal standard gene of experimental group). B = CT(control group target gene) − CT(control group reference gene), K = A − B, the fold change = 2^−K^.

## Results

### Morphological comparison between the retinal ganglion cell layer and nerve fiber layer of rats in each group

**Figure 1:**
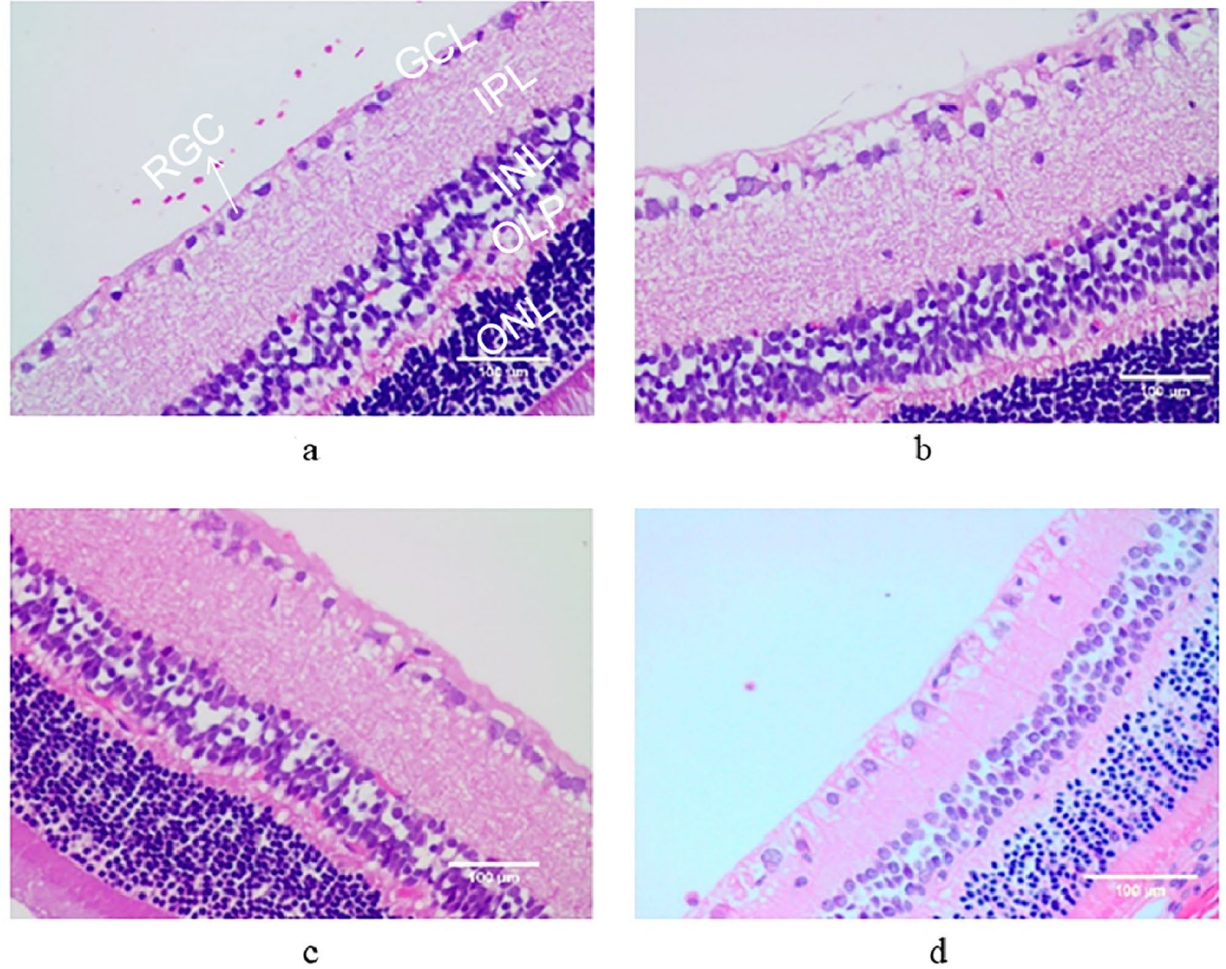
HE staining was performed in the normal control group (**a**), the simple ocular hypertension group (**b**), the 0.5% gallic acid group (**c**), and the 1% gallic acid group (**d**).

In the normal control group, the arrangement of cells in all layers was orderly, the RNFL structure was normal, the tissue was not edema, the size and shape of RGCs were similar, and the nucleus was clear (Fig. 1a). In the rats with simple ocular hypertension model, the RNFL tissues were loose in all layers of retinal edema, the RGCs were loosely arranged and the number was reduced, and some RGC nucleus were swollen and dissolved (Fig. 1b). The degree of RNFL edema in the 0.5% gallic acid group was significantly reduced compared with that in the simple high IOP group, and the arrangement of RGCs cells was more disordered in all levels, but the form of RGC was closer to normal than that in the simple high IOP group (Fig. 1c). Compared with the 0.5% gallic acid group, the RNFL tissues were slightly loose and edema was more obvious in the 1% gallic acid group, but the RGC cells were arranged slightly more neatly and their morphology was closer to normal in the 1% gallic acid group than in the 0.5% gallic acid group (Fig. 1d).

### Comparison of nerve fiber layer thickness in each group

RNFL thickness in the simple high IOP group and the gallic acid groups were significantly higher than that in the normal control group, and the total difference among the three groups was statistically significant (p < 0.01). The thickness of RNFL in the gallic acid group was lower than that in the simple high IOP group, the difference was statistically significant (p < 0.01) (Table 1).

**Table 1:** Comparison of nerve fiber layer thickness in each group.

Group	Samples	RNFL thickness	F	P
Normal control group	9	27.75 ± 9.49	53.162	< 0.001^a^
Simple ocular hypertension group	9	84.24 ± 7.31		0.0038^b^ 0.0017^c^
0.5% gallic acid experimental group	9	60.85 ± 1.79		–
1% gallic acid experimental group	9	53.26 ± 7.56		

Compared with the normal control group and the simple ocular hypertension group(^a^p < 0.01); compared with the simple ocular hypertension group and 0.5% gallic acid experimental group (^b^p < 0.01); compared with the simple ocular hypertension group and 1% gallic acid experimental group (^c^p < 0.01).

### ROS content in the retinal ganglion cell layer of rats in each group

**Figure 2:**
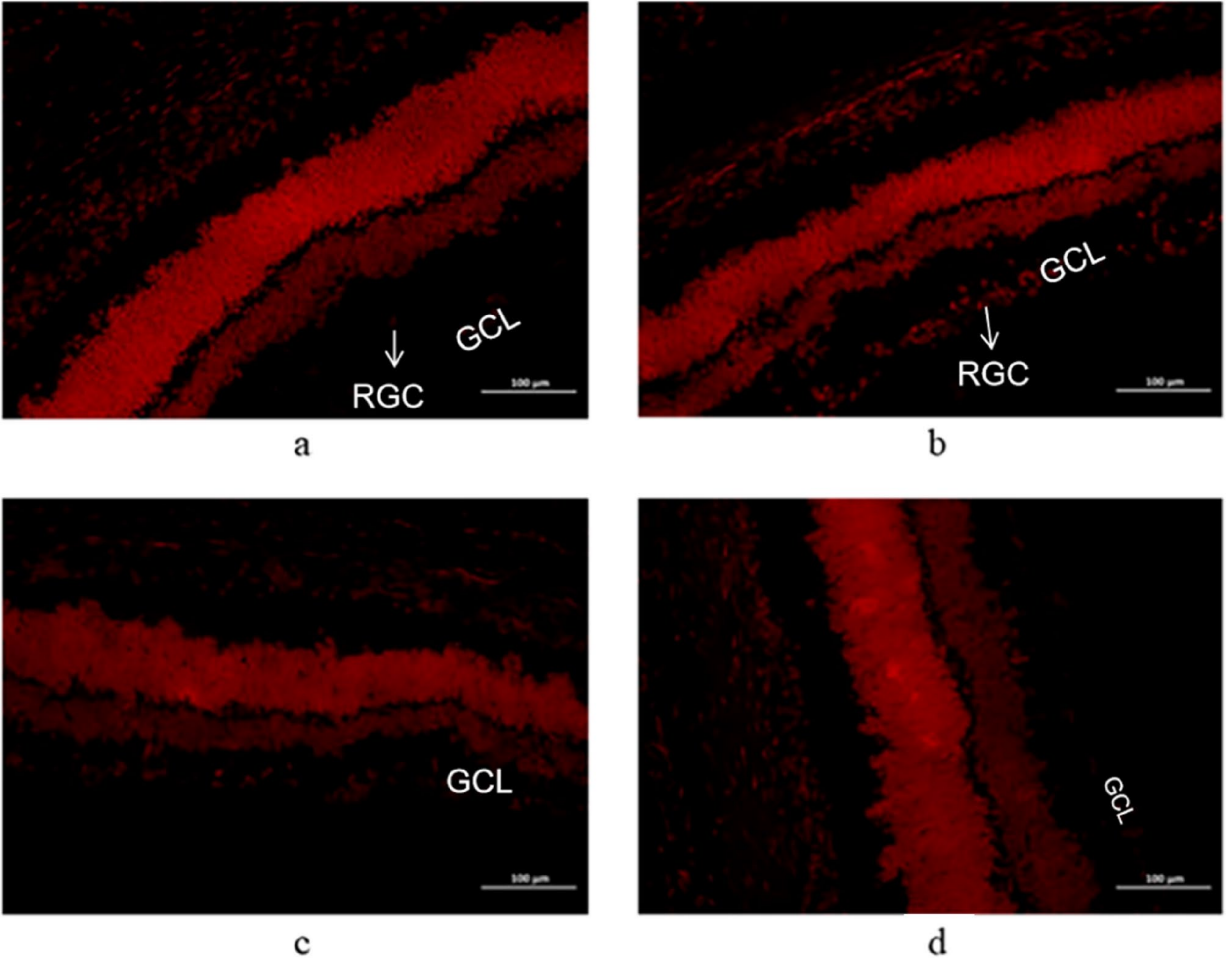
DHE staining was performed in the normal control group (**a**), the simple ocular hypertension group (**b**), the 0.5% gallic acid group (**c**), and the 1% gallic acid group (**d**).

**Figure 3:**
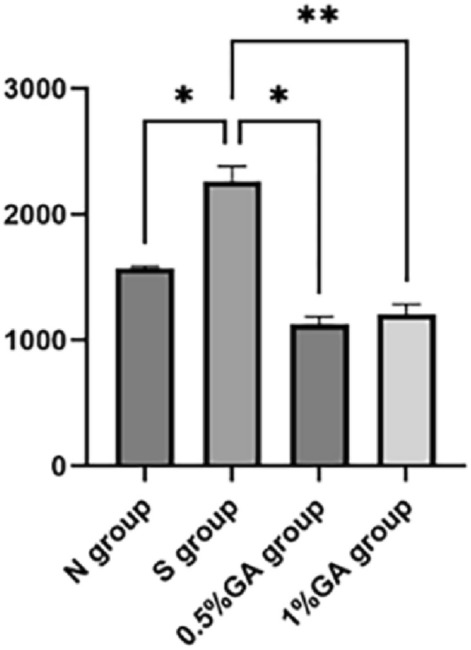
Comparison of the number of ROS-positive cells in the retina of each group. Compared to the normal control group, the simple ocular hypertension group had significantly increased ROS-positive cells (p < 0.05). Compared to the simple ocular hypertension group, the 0.5% GA group and 1% GA group had significantly decreased ROS-positive cells (p < 0.05).

The target area of the eye tissue is selected with a fluorescence photographic microscope for 200× imaging, and the image is filled with the entire field of view as much as possible to ensure that the background light is consistent in each photo. In the normal control group, DHE-contaminated ROS was almost invisible in the RGCs layer (Figs. 2a, 3), while in the simple ocular hypertension group, a large number of ROS were visible in the RGCs layer (Figs. 2b, 3), and in the 0.5% gallic acid group, the ROS content in the RGCs layer was significantly reduced compared with that in the simple high IOP group (p < 0.01) (Figs. 2c, 3, Table 2). The ROS content in the RGCs layer in the 1% gallic acid group was significantly decreased compared with that in the simple high IOP group alone (p < 0.01) (Figs. 2d, 3, Table 2).

**Table 2:** ROS-positive cell count in the retinal ganglion cell layer of rats in each group.

Group	Samples	Number of positive cells per unit area	F	P
Normal control group	9	1672.41 ± 130.59	12.005	0.013^a^
Simple ocular hypertension group	9	2261.21 ± 98.45		0.000799^b^ 0.000872^c^
0.5% gallic acid experimental group	9	1289.96 ± 277.28	–	–
1% gallic acid experimental group	9	1303.15 ± 186.22	–	

Compared with the normal control group and the simple ocular hypertension group (^a^*p* < 0.01); compared with the simple ocular hypertension group and 0.5% gallic acid experimental group (^b^*p* < 0.01); compared with the simple ocular hypertension group and 1% gallic acid experimental group(^c^*p* < 0.01).

### Count of retinal ganglion cells in each group

**Figure 4:**
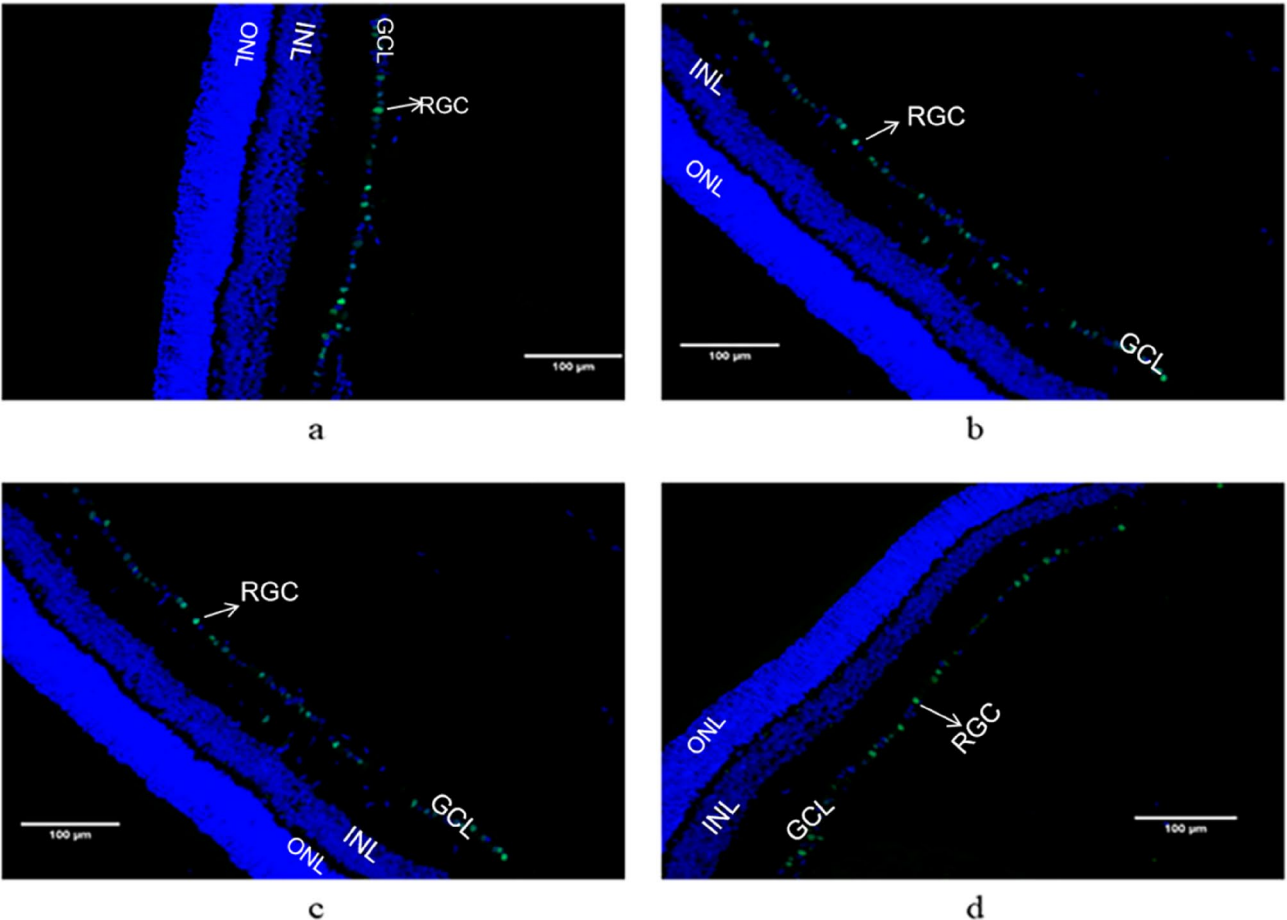
Immunofluorescent staining was performed in the normal control group (**a**), the simple ocular hypertension group (**b**), the 0.5% gallic acid group (**c**) and the 1% gallic acid group (**d**).

**Figure 5:**
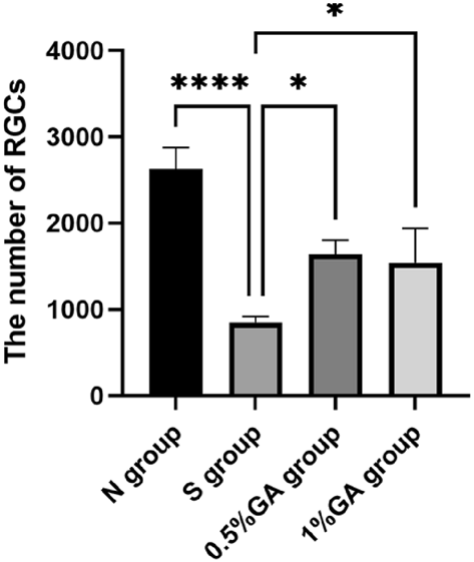
Comparison of the number of RGCs in the retina of each group. Compared to the normal control group, the simple ocular hypertension group had significantly decreased RGCs-positive cells (p < 0.05). Compared to the simple ocular hypertension group, the 0.5% and 1% GA groups had significantly increased RGCs-positive cells (p < 0.05).

The target area of rat retinal tissue was selected by fluorescence microscopy for 200-fold imaging. After the imaging was completed, the number of GCL layer positive cells in each section was measured using ImageJi53 analysis software. And the corresponding tissue area of the GCL layer (mm^2^), and calculated the number of positive cells per unit area (per mm^2^) = number of positive cells (per mm^2^)/tissue area of the GCL layer (mm^2^). SPSS26.0 software was used for analysis, and *P* < 0.05 was considered statistically significant. Antibody staining positive RGCs fluoresce green and cell nuclei fluoresce blue (DAPI). A large number of green fluorescent staining was observed in retinal ganglion cells of rats in the normal control group (Fig. 4a), while fewer green fluorescent cells were observed in the simple high IOP group compared with the normal control group (Fig. 4b). Compared with the simple high IOP group, more antibody-positive green fluorescent cells were found in the 0.5% gallic acid group and the 1% gallic acid group (Fig. 4c, d). The number of RGCs per unit area in the normal control group, the simple high IOP group, and the gallic acid experimental group were 2623.60 ± 208.59 (PCS), 864.81 ± 59.67 (PCS), 1636.92 ± 133.94 (PCS)and 1540.20 ± 326.55 (PCS) (Table 3). There were statistically significant differences between the normal control group and the simple high IOP group and between the the simple high IOP group and the gallic acid group (Fig. 5).

**Table 3:** Number of positive cells per unit area of retinal ganglion cells in rats.

Group	Samples	Number of positive cells per unit area	F	P
Normal control group	9	2623.60 ± 208.59	24.93	0.000026^a^
Simple ocular hypertension group	9	864.81 ± 59.67		0.005132^b^ 0.010122^c^
0.5% gallic acid experimental group	9	1636.92 ± 133.94		–
1% gallic acid experimental group	9	1540.20 ± 326.55		

Compared with the normal control group and the simple ocular hypertension group(^a^p < 0.01); compared with the simple ocular hypertension group and 0.5% gallic acid experimental group (^b^p < 0.01); compared with the simple ocular hypertension group and 1% gallic acid experimental group(^c^p < 0.01).

### The level of HIF-1α protein in the retina of rats in each group was detected by WB

**Figure 6:**
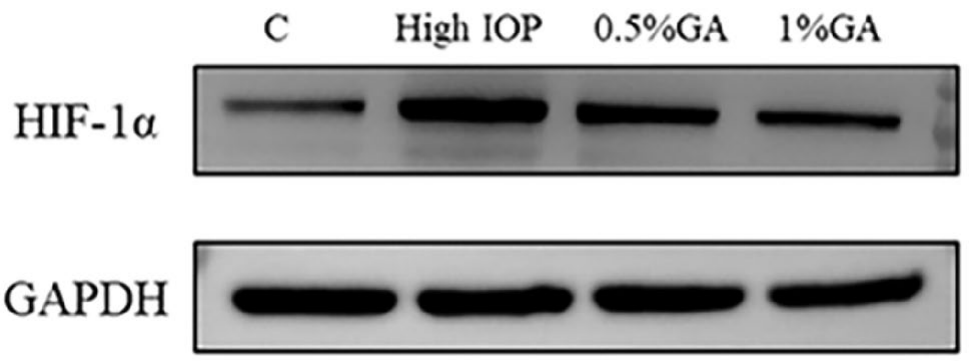
Western blot analysis of HIF-1α protein expression strip. C: the control group. High IOP: the simple ocular hypertension group. 0.5%GA: the 0.5% gallic acid group. 1%GA: the 1%gallic acid group.

**Figure 7:**
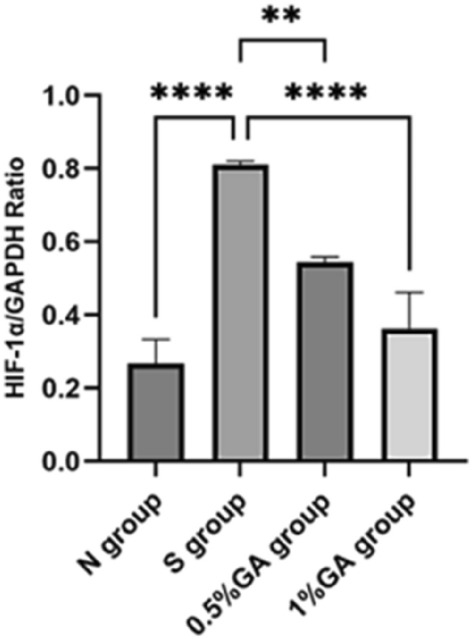
Western Blot analysis of HIF-1α/GAPDH ratio in each group. The simple ocular hypertension group had a significantly higher HIF-1α/GAPDH ratio than the normal control group (p < 0.05). The 0.5% and 1% GA groups had significantly lower HIF-1α/GAPDH ratios than the simple ocular hypertension group (p < 0.05).

The expression of HIF-1α protein in the retina of four groups (normal control group, the simple high IOP group, 0.5% gallic acid experimental group, 1% gallic acid experimental group) was detected by Western blot. Using GAPDH protein as an internal reference(Fig. 6), protein gray level analysis was used to calculate the relative expression level of each order protein. p < 0.05 was statistically significant(Fig. 7). As shown in Fig. 6, the expression level of HIF-1α protein in the simple high IOP group was significantly higher than in the normal control group and the gallic acid group. We used protein grayscale analysis to calculate the relative expression levels of the proteins in each group for statistical analysis. The results showed that the expression of HIF-1α protein could be increased under the condition of ischemia and hypoxia under ocular hypertension, while gallic acid could significantly inhibit the expression of HIF-1α protein.

### The HIF-1α RNA transcription level in the retina of rats in each group was detected by q-PCR

**Figure 8:**
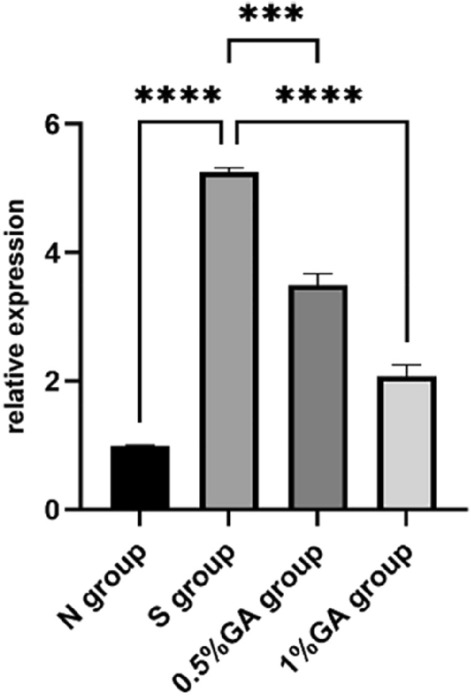
The relative expression of HIF-1α mRNA in each group was detected by qPCR. The simple ocular hypertension group had a significantly higher HIF-1α mRNA relative transcription level than the normal control group (p < 0.05). The 0.5% and 1% GA groups had significantly lower HIF-1α mRNA relative transcription levels than the simple ocular hypertension group (p < 0.05).

After sampling according to the method, the total RNA of the samples was extracted, and the mRNA expression of HIF-1α and GAPDH was detected by q-PCR. The relative transcription level of HIF-1α mRNA was significantly decreased after 0.5% gallic acid and 1% gallic acid application (p < 0.05)(Fig. 8). The primer sequences are as Table 4.

**Table 4:** The primer sequences as follows.

Primers	Base sequence (5′-3′)	Size of the product
R-GAPDH	Sense: CTGGAGAAACCTGCCAAGTATG	138
	Antisense: GGTGGAAGAATGGGAGTTGCT	
R-HIF-1α	Sense: GGCGAAGCAAAGAGTCTGAAGT	224
	Antisense: TAGCACCATAACAAAGCCATCC	

## Discussion

Glaucoma is a group of clinical syndromes characterized by optic nerve atrophy and visual field damage. Its pathological feature is the apoptosis of Retinal Ganglion Cells (RGCs) caused by increased intraocular pressure. The pathological increase of intraocular pressure is the main risk factor for glaucoma^12^. Reducing intraocular pressure cannot reverse the optic nerve damage and visual field loss caused by glaucoma^13^. In some patients, further optic atrophy occurs even when the intraocular pressure is reduced to an ideal state, eventually leading to tubular vision and even blindness. In addition to the pathological increase of intraocular pressure, glaucoma is a disease that involves multiple pathological factors^14^. Pathophysiological mechanisms of glaucoma include oxidative stress, inflammation, cell excitotoxicity, vascular damage and hypoxia, reactive glial cell activation, and axonal transport obstruction^15^.

In recent years, oxidative stress (OS) has been believed to be an important pathophysiological basis leading to optic nerve atrophy, and it is a phenomenon where the generation and elimination of free radicals are unbalanced. Highly active oxygen-containing molecules that cannot be removed are collectively known as Reactive Oxygen Species (ROS)^16^. When the eyeball is under harmful conditions such as ischemia and hypoxia caused by high intraocular pressure, a large number of superoxide ions (O2-) are generated, and a large number of ROS are generated under the action of superoxide dismutase (SOD)^17^. Firstly, oxidative stress stimulates the activation of the NF-κB pathway in trabecular reticulum cells, inducing mitochondrial damage of trabecular reticulum cells, which can also lead to apoptosis, change of trabecular reticulum structure, obstruction of aqueous reflux, resulting in the pathological increase of intraocular pressure, and eventually glaucoma^18^. In addition to the anatomical changes caused by damage to the trabecular network, the ensuing inflammatory response and the massive production of ROS can also cause oxidative damage to the optic nerve and cause glial dysfunction. ROS can be observed to trigger autophagy and apoptosis of RGCs in rat models of glaucoma, ultimately leading to optic nerve atrophy and blindness^19^. The increase of ROS content in cells will result in lipid peroxidation, and oxidative damage to proteins and DNA^20^.

Gallic acid (GA), also known as 3,4,5-trihydroxy benzoic acid (3,4,5-trihydroxy benzoic acid), is a natural secondary metabolic product. Gallic acid has one benzene ring, one carboxyl group, and three hydroxyl groups, and its unique chemical structure determines that it has powerful antioxidant functions^21^. It has been reported that the anti-oxidative stress function of gallic acid is inseparable from its unique chemical structure, and the phenolic hydroxyl group of gallic acid can provide hydrogen ions and ROS reactions generated during oxidative stress, remove ROS, and prevent the excessive formation of oxygen free radicals^22^. Nair. G et al. suggested that supplementation of 100 mg/kg gallic acid could reduce the whole-body DNA damage induced by gamma radiation in blood leukocytes, bone marrow cells, and spleen cells of irradiated mice^17^. The decrease of antioxidant GPX and GSH levels in γ-irradiated mice tissues can be recovered by supplementation of gallic acid and inhibition of lipid peroxidation, thus reducing the weight loss and mortality after γ-irradiated mice^23^. Gallic acid has also been shown to restore the antioxidant and inflammatory state to normal levels by activating the nuclear factor NRC-associated factor-2 (Nrf2) in the keap1-Nrf2-ARE pathway^24^. Gallic acid can also regulate the ERK/ NrF2-induced antioxidant signaling pathway and may have an antioxidant effect with Nrf2 competitive binding Keap1^25^. Liu et al. believed that gallic acid can inhibit oxidative stress by activating the Bax/Bcl-2 pathway, leading to hippocampus neurodegeneration in rats with type 2 diabetes^26^. In addition, in vitro antioxidant experiment results showed that gallic acid can effectively remove free radicals, hydroxyl radicals, and superoxide ion radicals, and has obvious inhibition on lipid peroxidation, with strong reducing power and total antioxidant power, and presents a good dose-dependent effect^6^. Gallic acid has a powerful antioxidant effect. At present, the antioxidant stress of gallic acid is mainly studied in liver cells, kidney cells, blood cells, and brain cells^27^. This experiment was designed to verify whether gallic acid has an antioxidant effect on retinal ganglion cells.

HIF-1α has been shown to increase immune markers in the optic nerve and retina in glaucoma compared to normal eyes. It has also been found that ROS is involved in the regulation of HIF-1α by intracellular silenced information regulatory factors. Mice with silenced information regulatory factor gene knockout showed increased ROS levels and HIF-1α expression, indicating that ROS is closely related to HIF-1α. Under hypoxia conditions, ROS production in cell mitochondria is induced and HIF-1α is activated. Gulgun et al. 's experiments showed that the expression of HIF-1α in the retina and optic nerve of glaucoma patients was significantly higher than that of normal people by immunohistochemistry, and the protein expression area even corresponds to the field of visual field defect, so they believed that retinal hypoxia in glaucoma may exist^28^.

Anterior chamber normal saline perfusion is to connect the infusion set to the sterile physiological salt bottle, then stab the needle into the anterior chamber of the rat, and raise the infusion bottle to a height of 150 cm above the level of the rat eyeball, which can increase the intraocular pressure by 110 mmHg, interrupt the retinal blood flow, and cause the apoptosis of retinal ganglion cells and nerve fiber edema^29^. The number of RGCs decreased, the content of ROS increased, and the content of HIF-1α increased. However, after the use of gallic acid, the neurofibroma decreased, the number of RGCs increased, the ROS content decreased significantly, and the protein level and transcription level of HIF-1α decreased.

In this experiment, gallic acid is a monomer component extracted from the Chinese herb gallnut, and the experimental results may indicate that gallic acid has a protective effect on the optic nerve of rats under the acute ocular hypertension model. At present, there are more and more traditional Chinese medicine treatments for glaucoma optic nerve damage, but most of the traditional Chinese medicine components are complicated and the mechanism is unknown, so it is necessary to continue to study the monomer drugs extracted from traditional Chinese medicine^30^. At present, the main problems in the research on the protective effect of traditional Chinese medicine and traditional Chinese medicine monomer on the optic nerve are reflected in the lack of a large number of clinical samples, even if its role is clearly defined in animal experiments, it is impossible to accurately evaluate its clinical efficacy.

Due to the lack of in vitro experiments of retinal ganglion cells in this study, the mechanism of action of gallic acid and the signaling pathway of oxidative stress should be further studied. Therefore, the exact mechanism of gallic acid protecting the optic nerve damaged by acute ocular hypertension needs to be further studied and perfected.

## Conclusions

In conclusion, we can draw the following conclusions: the acute ocular hypertension model in rats resulted in whole-layer retinal disorders, edema and thickness of retinal nerve fiber layer, swelling of RGCs nucleus, decrease of RGCs number, obvious apoptosis, sharp increase of ROS content, and high expression of HIF-1α in retinal ganglion cell layer in the acute ocular hypertension model. After the application of gallic acid, retinal nerve fiber layer edema was alleviated, which effectively alleviated the loss of RGCs caused by oxidative stress, decreased ROS content, and significantly decreased HIF-1α expression (Supplementary Information).

### Supplementary Information

Supplementary Information.

Supplementary Figures.

## Data Availability

The data that support the findings of this study are openly available. The datasets used and analysed during the current study available from the corresponding author on reasonable request.

## References

[1] Khatib TZ, Martin KR (2017). Protecting retinal ganglion cells. Eye.

[2] Wang W, He M, Li Z, Huang W (2019). Epidemiological variations and trends in health burden of glaucoma worldwide. Acta Ophthalmol. (Copenh.).

[3] Diekmann H, Fischer D (2013). Glaucoma and optic nerve repair. Cell Tissue Res..

[4] Hsueh Y-J (2022). The pathomechanism, antioxidant biomarkers, and treatment of oxidative stress-related eye diseases. Int. J. Mol. Sci..

[5] Xu Y, Tang G, Zhang C, Wang N, Feng Y (2021). Gallic acid and diabetes mellitus: Its association with oxidative stress. Molecules.

[6] Lu Y (2010). Gallic acid suppresses cell viability, proliferation, invasion and angiogenesis in human glioma cells. Eur. J. Pharmacol..

[7] Paraíso AF (2019). Oral gallic acid improves metabolic profile by modulating SIRT1 expression in obese mice brown adipose tissue: A molecular and bioinformatic approach. Life Sci..

[8] Lee J-W, Bae S-H, Jeong J-W, Kim S-H, Kim K-W (2004). Hypoxia-inducible factor (HIF-1)α: Its protein stability and biological functions. Exp. Mol. Med..

[9] Finley LWS, Haigis MC (2012). Metabolic regulation by SIRT3: Implications for tumorigenesis. Trends Mol. Med..

[10] Ergorul C (2010). Hypoxia inducible factor-1α (HIF-1α) and some HIF-1 target genes are elevated in experimental glaucoma. J. Mol. Neurosci..

[11] Gessi S (2010). Adenosine modulates HIF-1α, VEGF, IL-8, and foam cell formation in a human model of hypoxic foam cells. Arterioscler. Thromb. Vasc. Biol..

[12] Stein JD, Khawaja AP, Weizer JS (2021). Glaucoma in adults—Screening, diagnosis, and management: A review. JAMA.

[13] King AJ (2021). Primary trabeculectomy versus primary glaucoma eye drops for newly diagnosed advanced glaucoma: TAGS RCT. Health Technol. Assess..

[14] Tham Y-C (2014). Global prevalence of glaucoma and projections of glaucoma burden through 2040. Ophthalmology.

[15] Schuster AK, Erb C, Hoffmann EM, Dietlein T, Pfeiffer N (2020). The diagnosis and treatment of glaucoma. Dtsch. Ärztebl. Int..

[16] Milkovic L, Cipak Gasparovic A, Cindric M, Mouthuy P-A, Zarkovic N (2019). Short overview of ROS as cell function regulators and their implications in therapy concepts. Cells.

[17] Nita M, Grzybowski A (2016). The role of the reactive oxygen species and oxidative stress in the pathomechanism of the age-related ocular diseases and other pathologies of the anterior and posterior eye segments in adults. Oxid. Med. Cell. Longev..

[18] Gauthier AC, Liu J (2017). Epigenetics and signaling pathways in glaucoma. BioMed Res. Int..

[19] Weidinger A, Kozlov A (2015). Biological activities of reactive oxygen and nitrogen species: Oxidative stress versus signal transduction. Biomolecules.

[20] Razeghinejad R, Lin MM, Lee D, Katz LJ, Myers JS (2020). Pathophysiology and management of glaucoma and ocular hypertension related to trauma. Surv. Ophthalmol..

[21] Bai J (2021). Gallic acid: Pharmacological activities and molecular mechanisms involved in inflammation-related diseases. Biomed. Pharmacother..

[22] Sanz-Morello B (2021). Oxidative stress in optic neuropathies. Antioxidants.

[23] Nair GG, Nair CKK (2013). Radioprotective effects of gallic acid in mice. BioMed. Res. Int..

[24] Wang L (2011). Curcumin inhibits neuronal and vascular degeneration in retina after ischemia and reperfusion injury. PLoS ONE.

[25] Wei Y (2011). Nrf2 has a protective role against neuronal and capillary degeneration in retinal ischemia–reperfusion injury. Free Radic. Biol. Med..

[26] Liu Y, Carver JA, Calabrese AN, Pukala TL (2014). Gallic acid interacts with α-synuclein to prevent the structural collapse necessary for its aggregation. Biochim. Biophys. Acta BBA-Proteins Proteom..

[27] Verma S, Singh A, Mishra A (2013). Gallic acid: Molecular rival of cancer. Environ. Toxicol. Pharmacol..

[28] Tezel G (2004). Hypoxia-inducible factor 1α in the glaucomatous retina and optic nerve head. Arch. Ophthalmol..

[29] Overby DR, Clark AF (2015). Animal models of glucocorticoid-induced glaucoma. Exp. Eye Res..

[30] Garcia-Medina JJ (2020). Glaucoma and antioxidants: Review and update. Antioxidants.

