# Supplementary figures and images for: Antioxidant effect of gallic acid on retinal ganglion cells in glaucoma model
Source: Sci Rep. 2024 Jun 28;14:14907. doi: 10.1038/s41598-024-65965-7 (PMC11213916; doi:10.1038/s41598-024-65965-7)

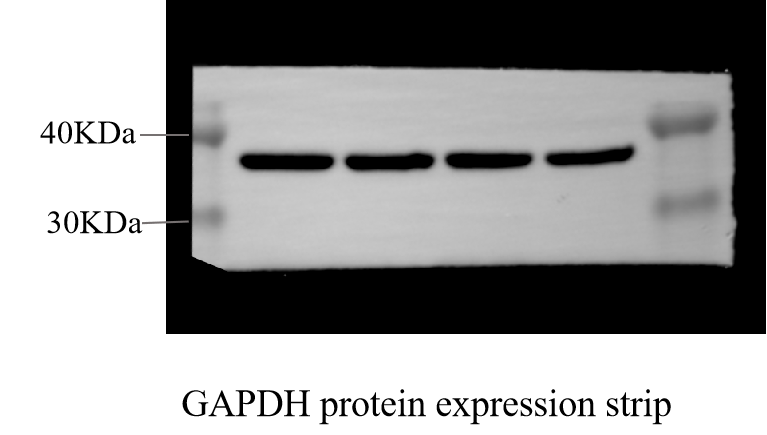

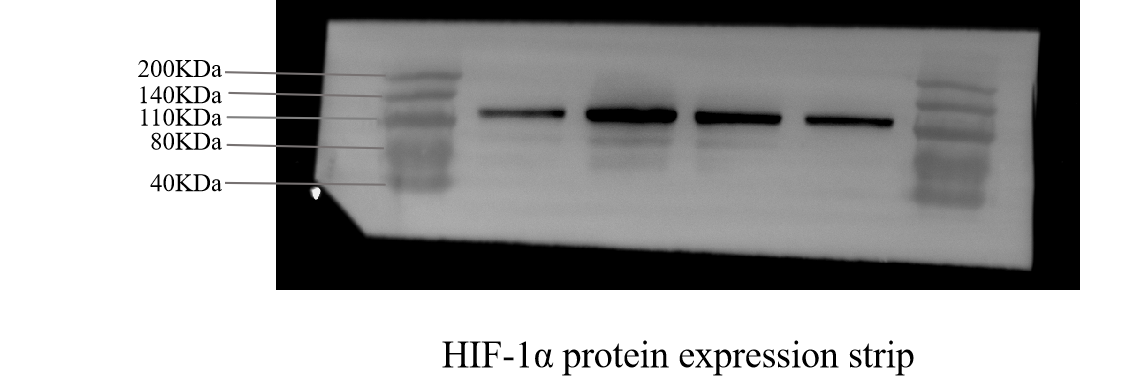

Supplement: Supplementary file 1 — Supplementary Information. [file 41598_2024_65965_MOESM1_ESM.docx]

figure1 GAPDH

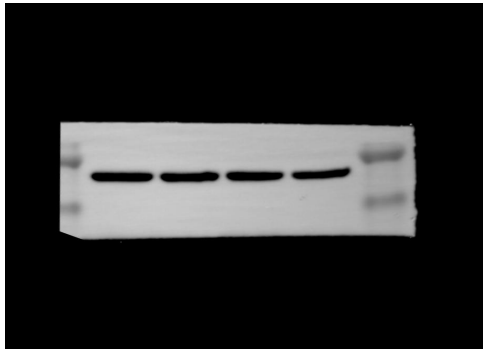

figure2 HIF-1α

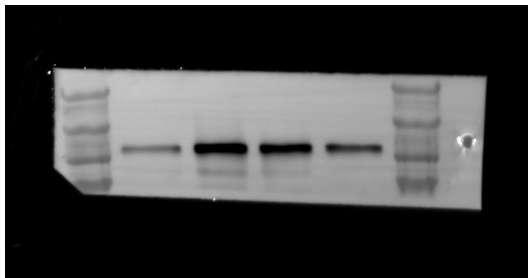

Supplement: Supplementary file 2 — Supplementary Figures. [file 41598_2024_65965_MOESM2_ESM.pdf]
